# Response of solar-induced chlorophyll fluorescence-based spatial and temporal evolution of vegetation in Xinjiang to multiscale drought

**DOI:** 10.3389/fpls.2024.1418396

**Published:** 2024-08-09

**Authors:** Cong Xue, Mei Zan, Yanlian Zhou, Zhizhong Chen, Jingjing Kong, Shunfa Yang, Lili Zhai, Jia Zhou

**Affiliations:** ^1^ School of Geographical Science and Tourism, Xinjiang Normal University, Urumqi, China; ^2^ Xinjiang Laboratory of Lake Environment and Resources in the Arid Zone, Urumqi, China; ^3^ School of Geography and Ocean Science, Nanjing University, Nanjing, China

**Keywords:** GOSIF, SPEI, multiscale, spatiotemporal characteristics, Xinjiang arid zone

## Abstract

Climate change and human activities have increased droughts, especially overgrazing and deforestation, which seriously threaten the balance of terrestrial ecosystems. The ecological carrying capacity and vegetation cover in the arid zone of Xinjiang, China, are generally low, necessitating research on vegetation response to drought in such arid regions. In this study, we analyzed the spatial and temporal characteristics of drought in Xinjiang from 2001 to 2020 and revealed the response mechanism of SIF to multi-timescale drought in different vegetation types using standardized precipitation evapotranspiration index (SPEI), solar-induced chlorophyll fluorescence (SIF), normalized difference vegetation index (NDVI), and enhanced vegetation index (EVI) data. We employed trend analysis, standardized anomaly index (SAI), Pearson correlation, and trend prediction techniques. Our investigation focused on the correlations between GOSIF (a new SIF product based on the Global Orbital Carbon Observatory-2), NDVI, and EVI with SPEI12 for different vegetation types over the past two decades. Additionally, we examined the sensitivities of vegetation GOSIF to various scales of SPEI in a typical drought year and predicted future drought trends in Xinjiang. The results revealed that the spatial distribution characteristics of GOSIF, normalized difference vegetation index (NDVI), and enhanced vegetation index (EVI) were consistent, with mean correlations with SPEI at 0.197, 0.156, and 0.128, respectively. GOSIF exhibited the strongest correlation with SPEI, reflecting the impact of drought stress on vegetation photosynthesis. Therefore, GOSIF proves advantageous for drought monitoring purposes. Most vegetation types showed a robust response of GOSIF to SPEI at a 9-month scale during a typical drought year, with grassland GOSIF being particularly sensitive to drought. Our trend predictions indicate a decreasing trend in GOSIF vegetation in Xinjiang, coupled with an increasing trend in drought. This study found that compared with that of the traditional greenness vegetation index, GOSIF has obvious advantages in monitoring drought in the arid zone of Xinjiang. Furthermore, it makes up for the lack of research on the mechanism of vegetation GOSIF response to drought on multiple timescales in the arid zone. These results provide strong theoretical support for investigating the monitoring, assessment, and prediction of vegetation response to drought in Xinjiang, which is vital for comprehending the mechanisms of carbon and water cycles in terrestrial ecosystems.

## 1 Introduction

Global warming is increasing the frequency of climate anomalies, with particular concern surrounding the increased occurrence of droughts (Liu et al., 2018b; Zuo et al., 2019; Zhong et al., 2023; Janni et al., 2024; Zhou et al., 2024). Droughts disrupt carbon and water cycle patterns in terrestrial ecosystems, primarily by impacting ecosystem composition, structure, and functioning. Piao et al. (2019) showed that prolonged drought and high temperatures may lead to soil nutrient loss, NPP reduction, and even vegetation death, with significant impacts on the global terrestrial carbon cycle. The incidence of drought disasters not only disturbs ecosystem balance but also poses a serious threat to socioeconomic well-being (Varela et al., 2019; Kaushik et al., 2023). Zhou et al. (2020) showed that drought in northeastern China has a significant impact on local maize yields, which are expected to reduce by 60% to 70% when water availability is halved. Therefore, studying the characteristics of vegetation response to drought holds substantial scientific value and practical significance. It furnishes a crucial scientific foundation for regional ecological environmental protection and sustainable economic development.

The aridity index serves as an effective method for assessing drought, simplifying the intricate phenomenon while gauging the severity of drought events (You et al., 2022). Numerous drought indices are currently utilized globally, regionally, and nationally. Among them, the Palmer drought severity index (PDSI) is widely used in long-term drought trend monitoring because of its comprehensive consideration of meteorological factors such as soil moisture and temperature (Jung-Ching et al., 2023), while the standardized precipitation index (SPI) is commonly used for short-term drought impact assessment because of its simplicity of calculation and consideration of precipitation only (Maedeh et al., 2023; Zhao et al., 2024). However, both of these indices have limitations in arid zones with complex and variable climates, especially in areas where droughts are frequent and of long duration. Among these, the PDSI cannot accurately reflect the dynamics of short-term droughts, and the SPI ignores the potential effects of climatic factors, such as temperature and wind speed, on droughts (Cao et al., 2023).

In the context of global warming, an increase in temperature and evapotranspiration has become a trend that cannot be ignored (Fang et al., 2019), and evapotranspiration has become crucial in drought and vegetation studies (Han et al., 2023). The standardized precipitation evapotranspiration index (SPEI) (Khalil et al., 2024; Marzieh and Rassoul, 2023) incorporates the contribution of heat factors to potential evapotranspiration based on the SPI (Xu et al., 2021a), rendering SPEI a more comprehensive and accurate measure of drought (Wang et al., 2020; Ma et al., 2023a). Additionally, SPEI offers greater flexibility than PDSI regarding timescales, adaptable to various requirements for drought monitoring (Anderson et al., 2011). As a result, SPEI holds evident advantages in drought monitoring and is now widely adopted in drought assessment research.

Numerous scholars have utilized various vegetation indices in exploring the mechanisms underlying vegetation responses to drought (Yang et al., 2018; Zhang et al., 2019). Normalized difference vegetation index (NDVI) is an early remote sensing vegetation index used to monitor the effects of drought on vegetation (AghaKouchak et al., 2015), which evaluates the electromagnetic spectrum of red and spectral features reflected in the near-infrared bands of the electromagnetic spectrum to assess vegetation growth. Doughty et al. (2021) showed that NDVI saturates with increasing LAI, whereas EVI improves the sensitivity and accuracy of vegetation monitoring by optimizing the ability to capture vegetation greenness signals (Aulia et al., 2016). While NDVI and EVI effectively monitor vegetation growth and “greenness,” they do not offer prompt responses to drought-induced alterations in vegetation photosynthesis (Song et al., 2018).

Furthermore, albedo-based vegetation indices suffer from the drawback of greenness saturation (Camps-Valls et al., 2021). Solar-induced chlorophyll fluorescence (SIF) is increasingly garnering attention from scholars as a novel indicator for monitoring vegetation growth (Guanter et al., 2007). Unlike traditional vegetation indices like NDVI and EVI, SIF can gauge the intensity of fluorescent signals emitted by vegetation during photosynthesis, offering a more direct and sensitive reflection of vegetation’s health status and photosynthetic capacity (Doughty et al., 2019). Mohammed et al. (2019) revealed that in the high vegetation cover area of tropical rainforest, SIF can more accurately reflect the real condition of vegetation growth compared with NDVI and EVI.

Many researchers have begun to explore the utilization of SIF for monitoring the impact of drought on vegetation. For instance, Jiao et al. (2019) discovered in their study that the sensitivity of SIF to drought varied among different vegetation types in different regions, with cultivated land exhibiting the highest sensitivity to short-term drought. Meanwhile, Jiang et al. (2021) identified a strong correlation between SIF and drought indices over long timescales. Additionally, Xu et al. (2021b) investigated the relationship between SIF and SPEI across various scales, observing variations in correlation based on timescale.

As the Xinjiang Uygur Autonomous Region stands as one of China’s most representative arid and semiarid regions, it possesses a fragile and intricate ecological environment. Despite this, recent studies in the region have predominantly relied on traditional greenness vegetation indices (Li et al., 2019; Yuan et al., 2021; Liu et al., 2022b), with only drought indices considering singular meteorological factors being employed in research on vegetation response to drought (Chen et al., 2015; Wu et al., 2022; Ruzi et al., 2023). In contrast, there is a paucity of investigations into drought monitoring and assessment utilizing SIF with SPEI across various timescales in Xinjiang’s arid region. Furthermore, there is a noticeable dearth of studies examining the response patterns of GOSIF across different vegetation types to SPEI during typical drought years, along with predictions regarding future SPEI trends in Xinjiang. Consequently, a lack of scientific consensus persists regarding the mechanism underlying vegetation GOSIF response to drought stress in Xinjiang’s arid zone.

In summary, this paper focuses on the arid region of Xinjiang and employs GOSIF and SPEI at various timescales to investigate the distinct responses of different vegetation types to drought in the area. Our study aimed to achieve the following objectives: determine trends in GOSIF and annual-scale SPEI from 2001 to 2020, along with changes in drought classification in the study area; evaluate the relationship between GOSIF and SPEI at different timescales for various vegetation types during a typical drought year in Xinjiang; and predict potential future changes in vegetation indicated by GOSIF and SPEI trends in Xinjiang. This study focuses on the applicability and superiority of GOSIF for drought monitoring in the arid zone of Xinjiang and attempts to determine the optimal timescale of vegetation GOSIF for drought monitoring in the study area. The results of this study fill the research gap regarding the response mechanism of vegetation GOSIF to drought in arid zones. The outcomes of these investigations not only contribute to ecological conservation efforts in Xinjiang but also offer valuable insights for global arid zone research.

## 2 Materials and methods

### 2.1 Overview of the study area

**Figure 1:**
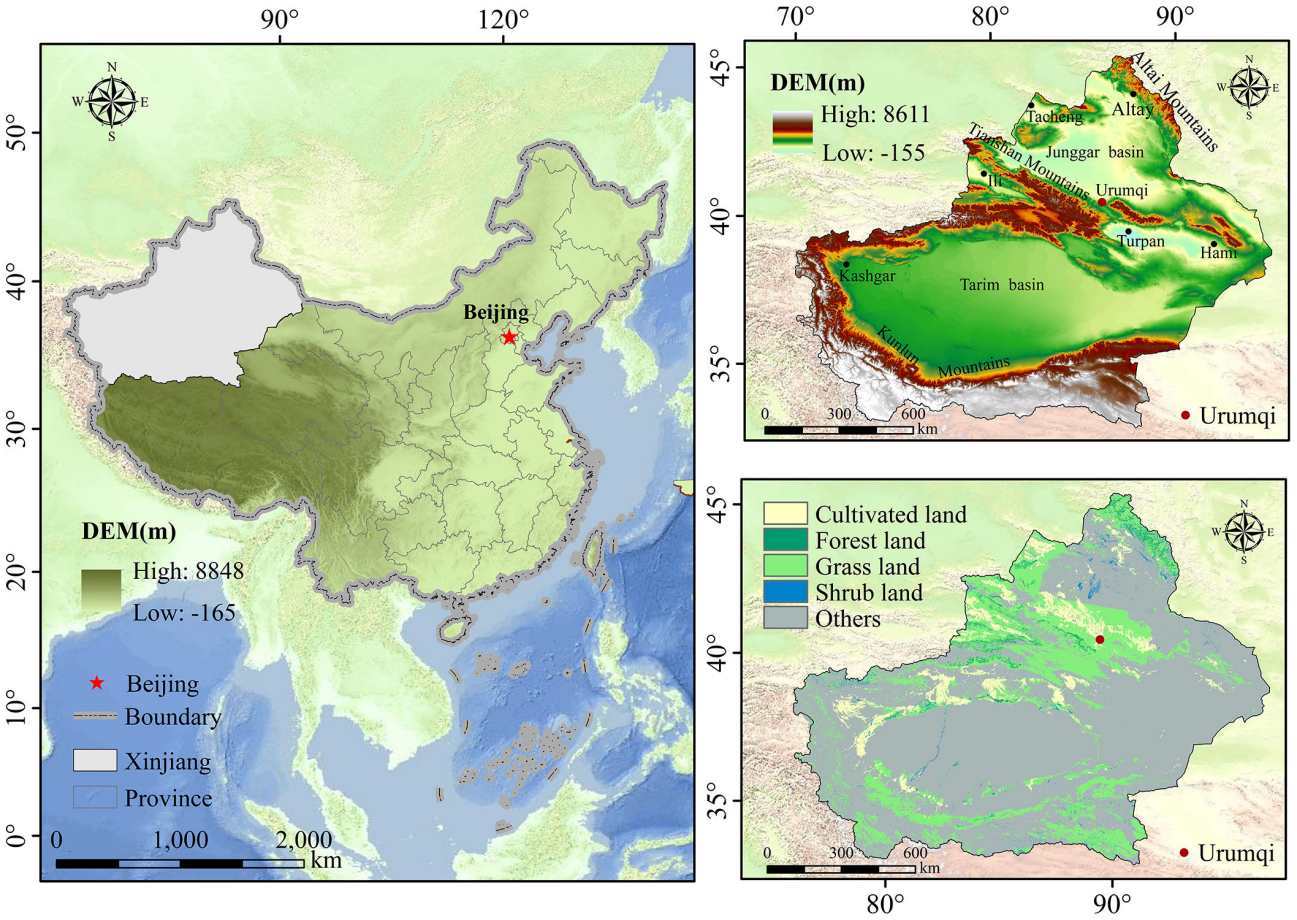
Overview of the study area.

Xinjiang is located in western China (73°40′–96°18′E, 34°25′–48°10′N), with a land area covering approximately one-sixth of the country’s surface area (**Figure 1**). Xinjiang, characterized as a classic arid and semiarid region, harbors fragile ecosystems (Song et al., 2019; Adila and Zan, 2022). Its topography is diverse, featuring a mosaic distribution of mountains, oases, and basins. Situated far from the sea and deeply inland, Xinjiang experiences a typical temperate continental climate marked by significant temperature variations, extended daylight hours, and pronounced evaporation effects. Vegetation cover in the region is sparse, comprising primarily cultivated land, forests, grasslands, and shrublands, with forests exhibiting the lowest coverage. Carbon stocks vary considerably among different vegetation types, with forests and grasslands boasting higher carbon stocks compared to other vegetation types. Rising temperatures lead to snow and ice melting, resulting in decreased water availability and exacerbated drought conditions. On average, Xinjiang witnesses approximately 0.5 drought disasters of varying severity annually, with a higher frequency of droughts observed in the Altay and Turpan regions. The period from 2001 to 2020 witnessed relatively severe drought conditions in Xinjiang, particularly in the Tarim Basin, Junggar Basin, and the northern slopes of the Tianshan Mountains, which experienced high incidences of drought disasters. Notably, severe drought events occurred in Xinjiang in 2006 and 2009, significantly impacting local agricultural production and the ecological environment.

### 2.2 Data sources and preprocessing

**Figure 2:**
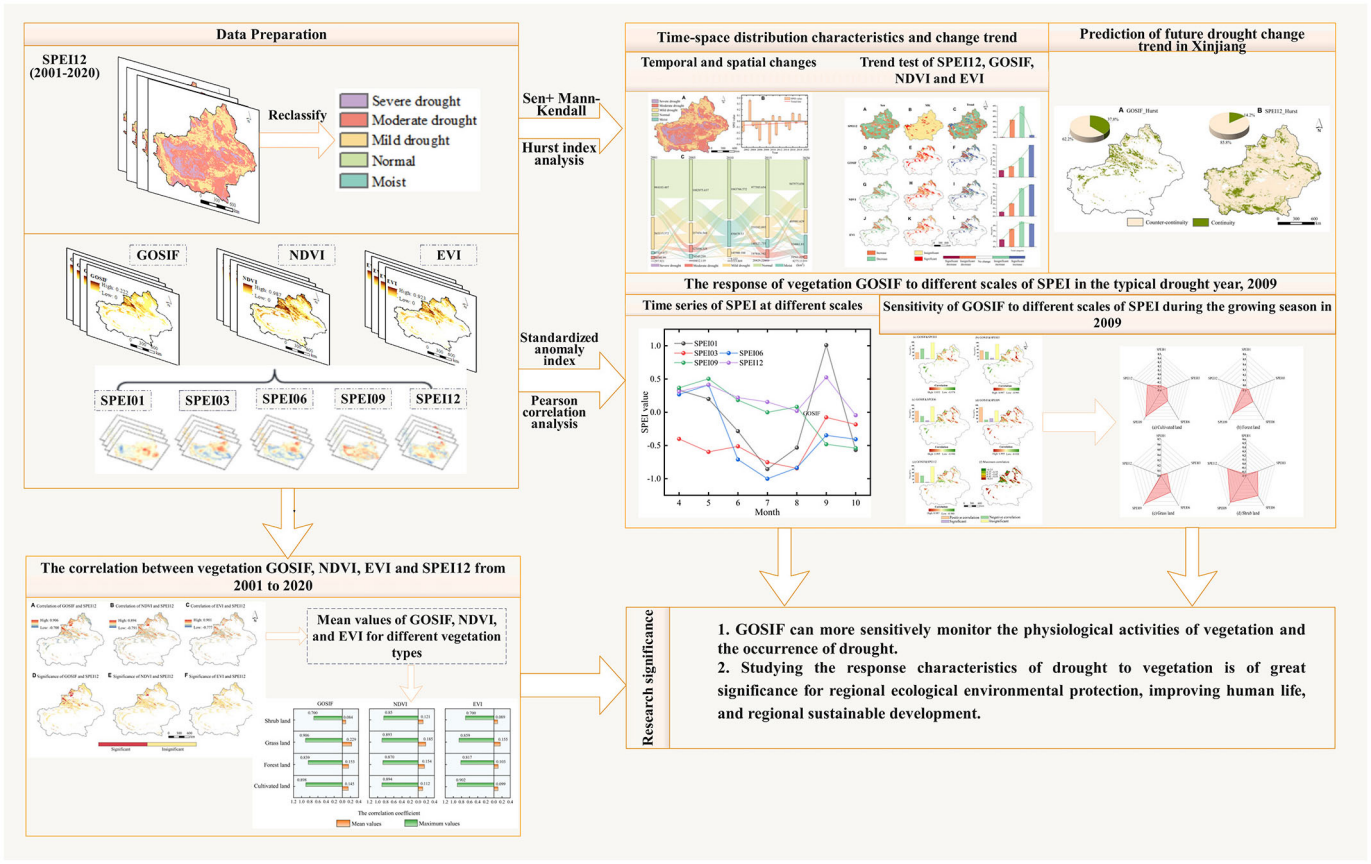
Technical framework.

In this study, we conducted a comprehensive analysis of the spatiotemporal characteristics between GOSIF and SPEI in the Xinjiang arid zone. We explored the sensitivities of GOSIF, NDVI, and EVI to multiscale SPEI, assessed their responses to drought across different vegetation types, and predicted the future trends of vegetation GOSIF and SPEI in Xinjiang. The research findings have provided theoretical evidence supporting the efficacy of vegetation GOSIF in monitoring drought in Xinjiang’s arid zone, thereby offering practical significance for ecological restoration and vegetation enhancement in the study area. The technical framework is depicted in **Figure 2**.

#### 2.2.1 GOSIF data

We utilized a global GOSIF dataset with high spatial and temporal resolution (0.05° × 0.05°, 8 days) generated by Li and Xiao (2019) based on discrete OCO-2 SIF sounding, MODIS remotely sensed, and meteorological reanalysis data (http://globalecology.unh.edu/data/GOSIF.html) as the solar-induced chlorophyll fluorescence product. GOSIF is characterized by finer spatial resolution and continuous global coverage compared to SIF, which is detected by OCO-2. We obtained GOSIF data for the Xinjiang region by cropping and resampling using the GOSIF annual-scale and 2009 monthly-scale products for China from 2001 to 2020.

#### 2.2.2 SPEI data

The SPEI data utilized in this study comprised a collection of raster datasets covering China from 2001 to 2020, featuring different timescales and high spatial resolution (1 km × 1 km). These datasets were calculated by Xia et al. (2023) using meteorological station data and employing the random forest method. Specifically, we selected SPEI12 (12-month scale) from 2001 to 2020 and SPEI01 (1-month scale), SPEI03 (3-month scale), SPEI06 (6-month scale), SPEI09 (9-month scale), and SPEI12 (12-month scale) data from 2009, which was deemed a typical anomalous drought year, for the study area across five scales. The SPEI drought classification is presented in **Table 1**.

**Table 1:** SPEI drought class classification.

Drought type	Extreme drought	Severe drought	Moderate drought	Mild drought	Normal	Moist
SPEI value	≤−2.0	−2.0 to −1.5	−1.5 to −1.0	−1.0 to −0.5	−0.5 to 0.5	≥0.5

To ensure consistency with the spatial resolution of the GOSIF data, the SPEI data, known for its highly consistent spatial and temporal distribution characteristics along with higher spatial resolution and improved drought identification compared to the widely recognized SPEI-based (v.2.6) dataset, were resampled to 0.05° × 0.05° (Xia et al., 2023).

#### 2.2.3 Vegetation index data

In this study, the 8-day scale MOD13A3 NDVI and EVI data from 2001 to 2020 were selected, and the 8-day scale NDVI and EVI were computed to obtain annual-scale counterparts using the maximum value synthesis (MVC) method on the Google Earth Engine (GEE) platform (Didan, 2015). Subsequently, we resampled the NDVI and EVI data to a spatial resolution of 0.05° × 0.05° to maintain consistency with our analysis.

### 2.3 Methods

#### 2.3.1 Trend analysis method

Theil-Sen is a statistical method for trend analysis of long-time series data (Equation 1), the calculation is as follows (Liu et al., 2016a; Kong et al., 2022).


(1) $$\omega =M\text{e}dian(\frac{x_{\text{b}}-x_{\text{a}}}{\text{b}-\text{a}})\;\forall \text{b}>\text{a}$$

The Mann–Kendall (MK) test, in combination with the Theil-Sen method, can reflect the overall trend of the data more accurately (Equations 2–5), avoiding the influence of errors to a large extent and improving the reliability of the results:


(2) $$T=\sum_{\text{a}=1}^{\text{k}-1}\sum_{\text{b}=\text{a}+1}^{\text{k}}\mathrm{sgn}(x_{\text{b}}-x_{\text{a}})$$

Sgn() is the sign function, which is calculated as follows:


(3) $$\text{sgn}(x_{\text{b}}-x_{a})=\{\begin{array}{ccc} +1 & & x_{\text{b}}-x_{a}>0\\ 0 & & x_{b}-x_{a}=0\\ -1 & & x_{b}-x_{a}<0 \end{array}$$

To perform the trend test, the test statistic *V* was calculated as follows:


(4) $$V=\{\begin{array}{ccc} \frac{T-1}{\sqrt{V\text{a}r(T)}} & & \left(T>0\right)\\ 0 & & \left(T=0\right)\\ \frac{T+1}{\sqrt{V\text{a}r(T)}} & & \left(T<0\right) \end{array}$$

Var was calculated as follows:


(5) $$V\text{a}r(T)=\frac{\left(\text{k}-1)(2\text{k}+5\right)}{20}$$

where ${\text{x}}_{\text{a}}$ and ${\text{x}}_{\text{b}}$ denote the image values of the image elements *a* and *b*, respectively, and k means the length of the time series, which was set as 20 in this study. When $│V│\leq V_{1-a/2}$ , the trend is insignificant; when $│V│\leq V_{1-a/2}$ , the trend is considered significant, and a significance level of α = 0.05 and a critical value $V_{1-a/2}$ = ± 1.96 were chosen. When the absolute value of *V* is greater than 1.65, 1.96, or 2.58, the trend passes the test of significance with a confidence level of 90%, 95%, or 99%, respectively (Tu et al., 2021). We superimposed the trends of GOSIF, SPEI12, NDVI, and EVI with the MK test results, and the resulting change trend was classified into five levels based on the slope values, namely, significant decrease, insignificant decrease, no change, insignificant increase, and significant increase.

#### 2.3.2 Standardized anomaly index

The SPEI trend was analyzed using linear regression, and the impact of drought on SPEI was characterized using the standardized anomaly index (SAI) (Equation 6) (Liu et al., 2016b; Kong et al., 2022):


(6) $$SAI_{FP}(\text{y})=\frac{FP(\text{y})-FP}{\sigma FP}$$

where SAI_FP_ (*y*) is the variance value of SPEI12 in year *y* (*y* = 1, 2, 3···20), FP (*y*) is SPEI12 in year *y*, FP denotes the mean value of SPEI12, and *σ*FP denotes the standard deviation of SPEI12. The SPEI12 standardized anomaly index is graded as normal (|SAI| ≤ 0.5), mildly abnormal (0.5 < |SAI| ≤ 1), moderately abnormal (1 < |SAI| ≤ 1.5), and severely abnormal (1.5 < |SAI| ≤ 2), and the drought conditions are more severe when the absolute value of SPEI12 is higher in the corresponding year.

#### 2.3.3 Correlation analysis

The Pearson correlation coefficient (Equation 7) is a value between [−1,1], and the closer its absolute value is to 1 or −1, the stronger the linear relationship between the two variables and the stronger the sensitivity between the two variables (Shi et al., 2002). If the coefficient is positive, it represents a positive correlation, and if the coefficient is negative, it represents a negative correlation. The correlation coefficient between two variables is represented by Rxy with the following formula (Qi et al., 2023).


(7) $$R_{xy}=\frac{\sum_{\text{a}=1}^{\text{m}}\left[(x_{\text{a}}-\bar{x})(y_{\text{a}}-\bar{y})\right]}{\sqrt{\sum_{\text{a}=1}^{\text{m}}{\left(x_{\text{a}}-\bar{x}\right)}^{2}}\sqrt{\sum_{\text{a}=1}^{\text{m}}{\left(y_{\text{a}}-\bar{y}\right)}^{2}}}$$

where m is the number of time series (20), and $\bar{\text{x}}$ and $\bar{\text{y}}$ are the mean values of the variables. In this paper, Pearson correlation was used to analyze the relationship between SPEI12 and GOSIF and NDVI and EVI from 2001 to 2020, as well as the correlation between GOSIF and SPEI at different scales during the 2009 growing season.

#### 2.3.4 Hurst index analysis

The Hurst index *H* is used to predict whether GOSIF and SPEI in Xinjiang will maintain the past trend in the future (Equations 8–11). The calculation formula is as follows.


(8) $$X(t,\gamma )=\sum_{t=1}^{t}\left(SPEI(t)-\bar{SPEI}(\gamma )\right)\begin{array}{cc} & 1\leq \text{t}\leq \gamma \end{array}$$

(9) $$R\left(\gamma \right)=\underset{1\leq \text{t}\leq \gamma }{\max X(t,\gamma )}-\underset{1\leq \text{t}\leq \gamma }{\min X(t,\gamma )}\begin{array}{cc} & \gamma =1,2,\cdot \cdot \cdot ,n \end{array}$$

(10) $$S(\gamma )={\left[\frac{1}{\gamma }\sum_{t=1}^{\gamma }{\left(SPEI(\text{t})-SPEI(\gamma )\right)}^{2}\right]}^{\frac{1}{2}}\;\gamma =1,2,\cdot \cdot \cdot ,n$$

Where *X*(*t,γ*) is the cumulative deviation series, *R*(*γ*) is the extreme deviation series, *S*(*γ*) is the standard deviation series, and SPEI(*t*) and SPEI(*γ*) denote SPEI data for year *t* and year *γ*. We used the ratio of *R*(*γ*) to *S*(*γ*) to invert the Hurst index, as follows (Tong et al., 2018).


(11) $$R/S=R(\gamma )/S(\gamma )={\left(\frac{\pi \gamma }{2}\right)}^{H}$$

Where *H* is the Hurst index and the value domain of *H* is [0, 1]. When 0 ≤ *H* < 0.5, it means that the future GOSIF or SPEI in Xinjiang may be opposite to the past trend; when 0.5 < *H* ≤ 1, it means that the future GOSIF or SPEI in Xinjiang may continue to maintain the past trend; and when *H* = 0.5, it means that there is no clear correlation between the future trend and the past (Kong et al., 2022).

## 3 Results

### 3.1 Characteristics of spatial and temporal distribution and trends of SPEI12, GOSIF, NDVI, and EVI

**Figure 3:**
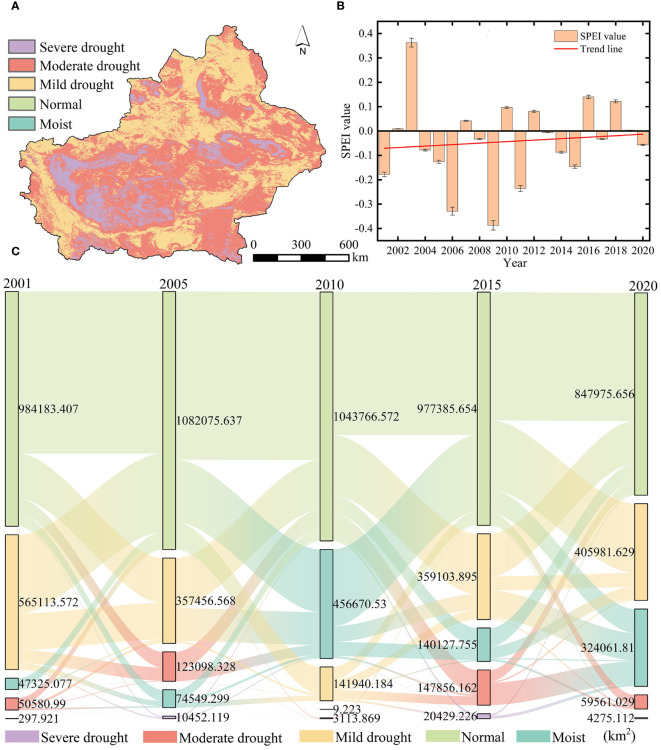
**(A)** The synthetic spatial distribution of year-scale SPEI12 minima during 2001–2020. **(B)** The time series of 2001–2020 SPEI12 means. **(C)** The shifts in annual-scale drought levels during 2001–2020.

The spatial distribution of the drought classification of the 2001–2020 SPEI12 minima in Xinjiang (**Figure 3A**) reveals that moderate drought predominantly covered 53.07% of the total study region, spanning the eastern part of Xinjiang, the Junggar Basin, the Altai Mountains, and the vicinity of the Tarim Basin. Additionally, 10.44% of severe drought was concentrated in the southwestern part of the Tarim Basin. Mild drought primarily occurred along the northern portion of Tacheng, the Tianshan Mountains, and the Kunlun Mountains region. Analyzing the SPEI12 trend over the last 20 years across the study region (**Figure 3B**), we observed that the mean value of SPEI12 was the lowest in 2009 and the highest in 2003. For the last two decades, drought conditions in the study area gradually diminished, transitioning toward progressively wetter conditions. Examining the transfer of different drought classes of SPEI12 in the study area in 2001, 2005, 2010, 2015, and 2020 (**Figure 3C**), we noted a decreasing tendency in moist and mild drought areas, while areas classified as normal or severely and moderately drought-ridden displayed an increasing tendency. However, overall, the areas experienced a gradual shift toward wetter conditions. The sums of the areas where all drought types changed between the different years 2001–2005, 2005–2010, 2010–2015, and 2015–2020 were 4.154 × 10^5^ km², 7.178 × 10^5^ km², 7.652 × 10^5^ km², and 4.640 × 10^5^ km², respectively. Over the past 20 years, the rise in mild drought within the study area mainly stemmed from the conversion of regions that were initially moist or normal. Meanwhile, the escalation of moderate drought primarily occurred in areas previously characterized as moist, normal, or experiencing mild drought conditions.

The area that changed from moist to mildly and moderately arid in the last 20 years spanned 0.146 × 10^5^ km² and 0.018 × 10^5^ km², respectively. Moderate drought during 2001–2005 and 2010–2015 primarily occurred in moist, normal, and mild drought areas covering areas of 1.207 × 10^5^ km² and 1.478 × 10^5^ km², respectively. Compared to that in 2001, the area in the moist and normal classes increased by 2.224 × 10^5^ km² in 2020, suggesting that drought conditions in Xinjiang have been alleviated to some extent in the last 20 years.

**Figure 4:**
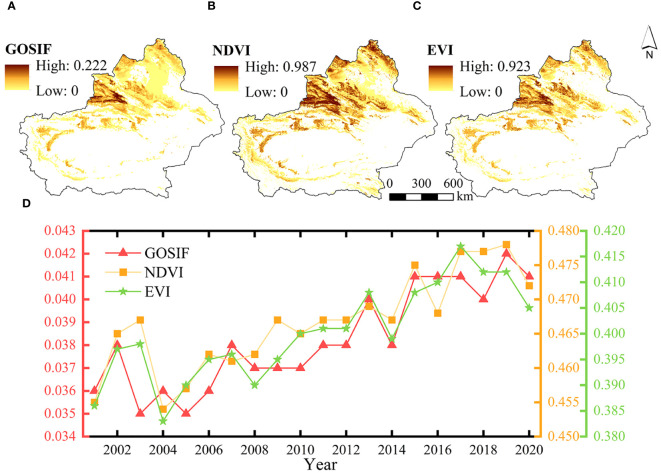
The spatial distribution of multiyear means of GOSIF **(A)**, NDVI **(B)**, and EVI **(C, D)** a time series of GOSIF, NDVI, and EVI changes from 2001 to 2020.

Spatial distribution of the mean values of vegetation GOSIF, NDVI, and EVI from 2001 to 2020 in the study area (**Figures 4A–C**) indicates that the spatial distribution of the three indices has a good consistency and the high values are largely distributed in areas around the Ili River Valley. Among them, high NDVI values were concentrated in the Altai Mountains, Ili region, and Tianshan Mountains. As depicted by the curves illustrating the mean values of GOSIF, NDVI, and EVI for vegetation in the study area over the last 20 years (**Figure 4D**), the overall variation trend was largely similar across all three indices, exhibiting clear fluctuations and increasing tendencies. Specifically, the mean value of GOSIF peaked in 2019 at 0.042, with a rate of increase of 0.0003 per annum. Similarly, the mean value of NDVI also reached its highest point in 2019, while the mean value of EVI achieved its maximum in 2016. Collectively, these findings suggest a gradual enhancement in the photosynthetic capacity and greenness of vegetation in the arid zone of Xinjiang during the period from 2001 to 2020, accompanied by a partial alleviation of drought conditions.

**Figure 5:**
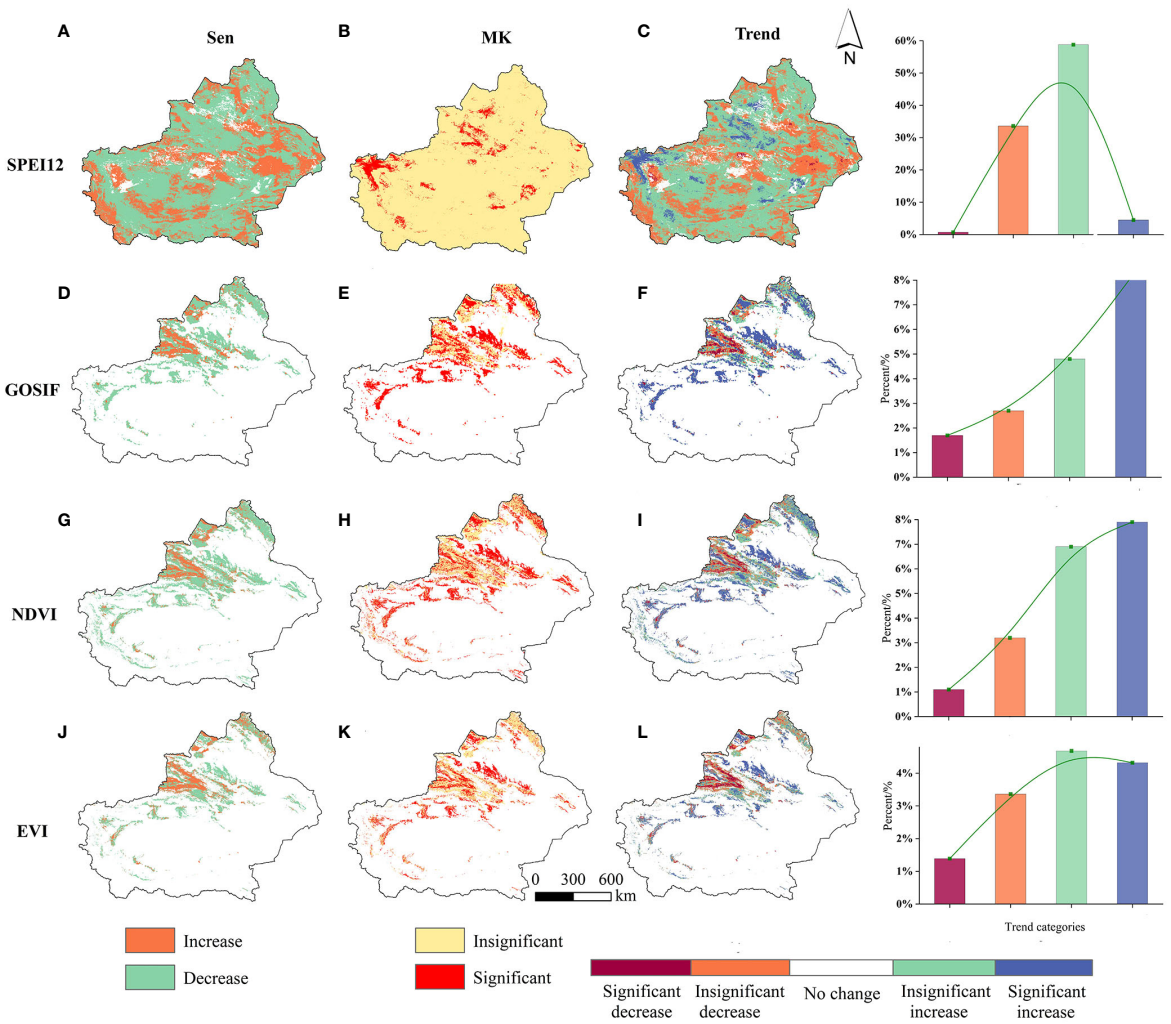
The spatial distribution of SPEI12, GOSIF, NDVI, and EVI trends **(A, D, G, J)** and significance tests **(B, E, H, K)**; **(C, F, I, L)** in the five classes, namely, significant decrease, insignificant decrease, no change, insignificant increase, and significant increase spatial distributions.

We used the Sen+Mann–Kendal method to analyze the trends and significance of SPEI12, GOSIF, NDVI, and EVI in Xinjiang from 2001 to 2020. As illustrated in **Figure 5**, all four variables—SPEI12, GOSIF, NDVI, and EVI—exhibited increasing trends, with the area of increase surpassing that of decrease (**Figures 5A, D, G, J**). Among them, the areas with significant increases (*p* < 0.05) in SPEI12 accounted for 4.55% of the total study area, which was mainly distributed in the eastern part of Ili Valley, southeastern part of Tacheng, and central part of Bazhou, suggesting progressive wetness in these areas (**Figures 5B, C**). The areas where the trends of GOSIF, NDVI, and EVI changes passed the 95% significance test were mainly in the Altai Mountains, Tacheng, Ili, and the northern slope of the Tianshan Mountains (**Figures 5E, H, K**). Among them, the percentages of areas with significant increases in GOSIF, NDVI, and EVI in the total study area were 8.15%, 8.03%, and 4.32%, respectively (**Figures 5F, I, L**), whereas southwest Tacheng and the vicinity of the southern slope of the Tianshan Mountains showed a significant decreasing trend in GOSIF, NDVI, and EVI. The significant increase in GOSIF, NDVI, and EVI of vegetation in Xinjiang over the last 20 years indicated a gradual improvement in vegetation productivity and cover in the study area. These changes not only help improve the carbon storage capacity but also are important for promoting the sustainable development of vegetation resources in the study area.

### 3.2 Correlation of annual-scale vegetation GOSIF, NDVI, and EVI with SPEI12

**Figure 6:**
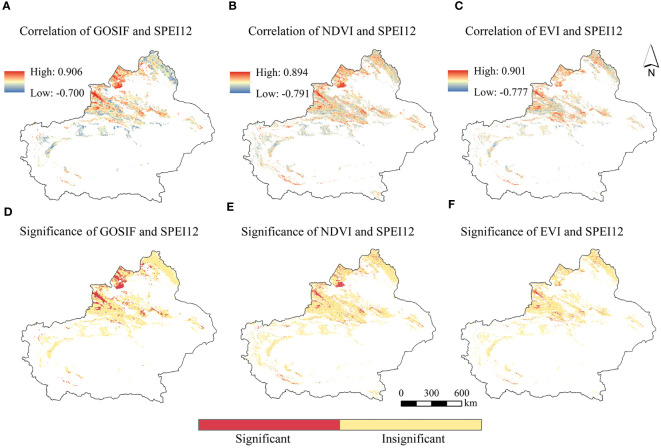
The spatial distribution of GOSIF, NDVI, EVI, and SPEI12 correlations **(A–E)** and significance **(D–F)**.

From the correlation analysis of vegetation GOSIF, NDVI, and EVI with SPEI12 in Xinjiang over the past 20 years (**Figure 6**), it is evident that the correlations of all three variables were consistently positive across the spatial domain. The results showed a close correlation between vegetation growth conditions and the degree of drought. When the value of SPEI12 decreased, meaning that the degree of drought increased, vegetation growth was stressed by drought, and the values of GOSIF, NDVI, and EVI decreased accordingly. The mean correlation values of GOSIF, NDVI, and EVI with SPEI12 were 0.197, 0.156, and 0.128, respectively, with their maximum values reaching 0.906, 0.894, and 0.902, respectively. Moreover, the proportions of GOSIF pixels significantly and positively correlated (*p* < 0.05) with SPEI12, as well as NDVI and EVI pixels significantly correlated (*p* < 0.05) with SPEI12, accounting for 11.73%, 8.40%, and 7.12% of the total study area, respectively. The results suggest that these indices can serve as important indicators for drought monitoring and environmental assessment in the study area. Notably, pixels significantly correlated with SPEI12 by GOSIF and NDVI were predominantly situated in the southern part of Tacheng and the northern part of Ili. Conversely, EVI exhibited significant correlation mainly in the Ili region. GOSIF demonstrated a higher correlation with SPEI12 and appeared more sensitive to drought compared to NDVI or EVI. This indicates that, for arid regions, GOSIF shows clear advantages in drought monitoring and assessment.

**Figure 7:**
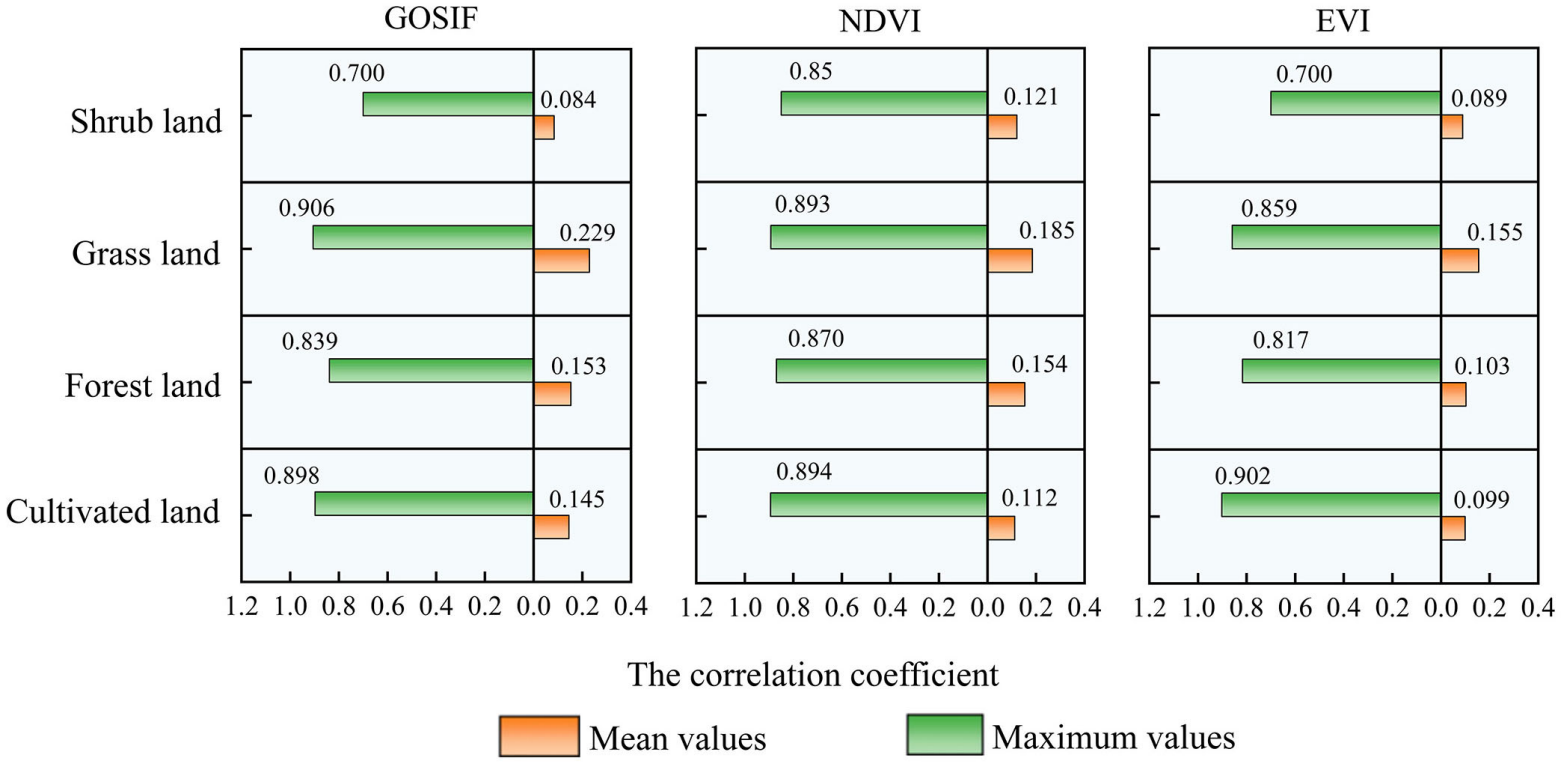
The mean and maximum values of EVI, NDVI, and GOSIF for different vegetation types on an annual scale.

Among the different vegetation types, the mean correlation coefficients of grassland for GOSIF, NDVI, and EVI with SPEI12 were the highest (**Figure 7**). Specifically, the mean correlation of NDVI with SPEI12 for forest and shrubland exceeded that of GOSIF and EVI with SPEI12. However, the mean correlation of GOSIF with SPEI12 for forestland was only marginally lower than that of NDVI with SPEI12.

Notably, both the average and maximum correlation coefficients between grassland GOSIF and SPEI12 were notably higher, reaching 0.229 and 0.906, respectively, surpassing the corresponding correlation coefficients of NDVI and EVI with SPEI12. **Figures 1**, **6**, **7** collectively illustrate that the high correlation values of GOSIF, NDVI, and EVI with SPEI12 for grassland were predominantly concentrated in the Tacheng and Ili regions, with correlations diminishing toward the periphery of the Tarim Basin. Among all vegetation types, shrubland exhibited the lowest correlation of GOSIF, NDVI, and EVI with SPEI12. Consequently, GOSIF emerged as a practical and superior indicator for assessing drought responses.

### 3.3 Response of GOSIF to different scales of SPEI in a typical drought year

#### 3.3.1 Sensitivity of growing season GOSIF to SPEI at different scales

**Figure 8:**
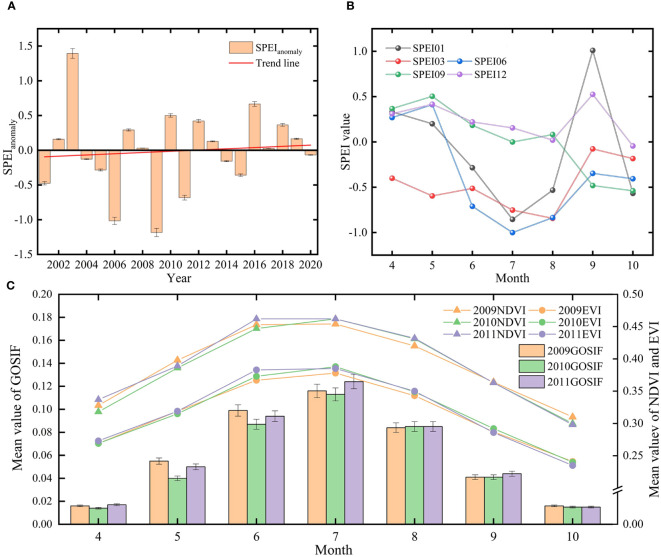
**(A)** The standardized anomaly index (SPEI) from 2001 to 2020. **(B)** The trend of SPEI at different scales during the growing season of 2009. **(C)** The mean values of GOSIF, NDVI, and EVI during the growing season from 2009 to 2011.

Based on the definition of moderately anomalous years by the SAI (1 < |SPEI*_anomaly_* | < 1.5), the moderately anomalous years of the SPEI in the study area were identified as 2003, 2006, and 2009 (**Figure 8A**). When considering the trend of annual-scale changes in the SPEI12 from 2001 to 2020 in the study area, it became apparent that 2009 stood out as the most typical anomalous drought year. Therefore, we further analyzed GOSIF with SPEI at different scales during the 2009 vegetation growing season (April–October). From the mean values of different scales of SPEI (**Figure 8B**), the mean value of SPEI06 in July was −1.01, indicating moderate drought; the area was relatively moist in September, whereas SPEI01 and SPEI03 in July indicated mild drought, with the mean values of −0.855 and −0.753, respectively; and SPEI09 and SPEI12 of the growing season indicated normal conditions, overall.

From **Figure 8C**, it is evident that compared to the 2009 growing season, the mean values of GOSIF, NDVI, and EVI exhibited a decreasing trend in the 2010 growing season followed by an increasing trend in the 2011 growing season, thereby affirming the classification of 2009 as a typical drought year. Due to the lag effect of vegetation on the impact of drought, following the occurrence of drought in 2009, the GOSIF of vegetation during the growing season of 2010, particularly in April–June, experienced a rapid decline with a rate of decline of 16.667%. In contrast, the rate of decline of NDVI was only 2.333%, while EVI began to show a declining trend only in May. Vegetation growth resumed from April to June 2011, during which GOSIF, NDVI, and EVI exhibited an increasing trend. Notably, the average growth rate of GOSIF reached 17.133%, significantly surpassing the growth rates of NDVI (3.351%) and EVI (1.827%).

In summary, both the impact period following drought onset and the recovery period after drought highlighted the rapid increase and decrease characteristics of vegetation GOSIF in response to drought impacts when compared to NDVI and EVI.

**Figure 9:**
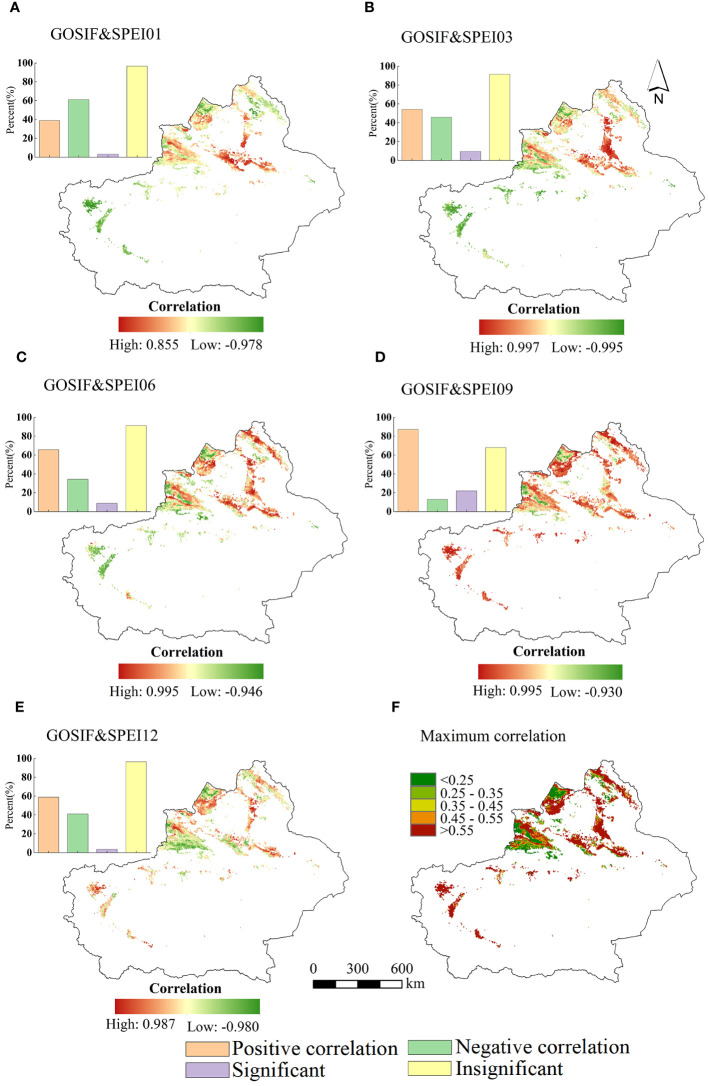
The spatial distribution of the correlation of GOSIF with SPEI01 **(A)**, SPEI03 **(B)**, SPEI06 **(C)**, SPEI09 **(D)**, and SPEI12 **(E)** for vegetation during the growing season of 2009. **(F)** The spatial distribution of the maximum synthetic value of the correlation coefficients of GOSIF and the multiscale SPEI.

From the spatial distribution of GOSIF with different scales of SPEI during the growing season (**Figures 9A–E**), it is evident that GOSIF exhibited the highest mean correlation (0.460) with SPEI09, followed by SPEI06, while the lowest correlation was observed with SPEI01, displaying an overall weak negative correlation. **Figure 9E** illustrates that approximately 90.050% of the total vegetation area in Xinjiang exhibited a positive correlation between GOSIF and SPEI, with 22.070% of the vegetation areas passing the 95% significance test.

Analyzing the spatial distribution of the maximum values of the correlation coefficients between vegetation GOSIF and SPEI at different timescales (**Figure 9F**), it can be observed that the maximum correlation coefficients ranged from 0.450 to 0.997, with a mean value of the maximum positive correlation coefficients estimated at 0.535. Areas with correlation coefficients greater than 0.550 were concentrated in the Altai Mountains, southern Tacheng, northern slopes of the Tianshan Mountains, and the northwestern Tarim Basin. Meanwhile, areas with positive correlation coefficients ranging between 0.450 and 0.550 were primarily distributed in the Ili region. Additionally, regions displaying a negative correlation between GOSIF and SPEI were observed within the Ili Valley.

#### 3.3.2 GOSIF response to SPEI for different vegetation types

**Figure 10:**
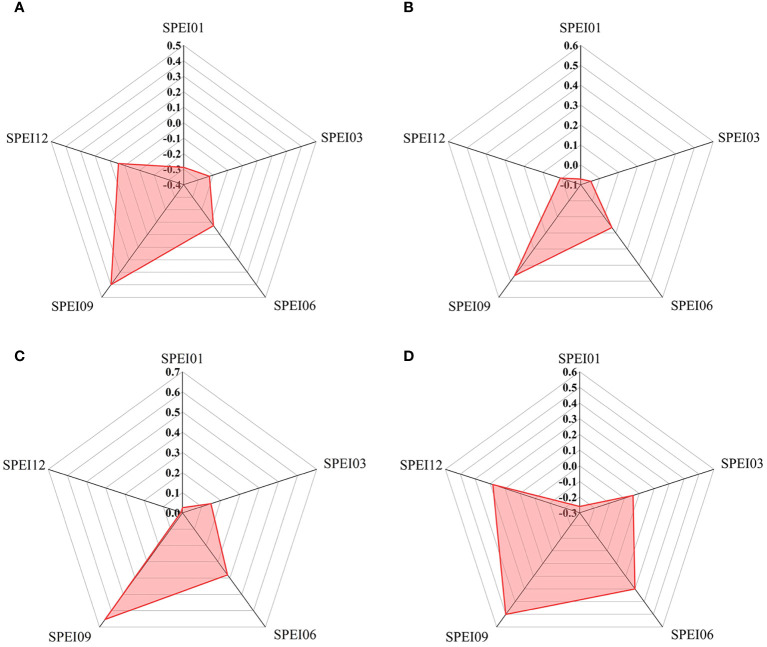
The mean values of correlation coefficients between GOSIF and SPEI at different scales in 2009 for cultivated land **(A)**, forestland **(B)**, grassland **(C)**, and shrubland **(D)**.

The mean correlation coefficients between GOSIF and SPEI at different timescales during the growing season (**Figure 10**) indicate that, for most vegetation in the study area, the largest mean correlation coefficients were observed between GOSIF and SPEI09. On average, the correlation coefficients between GOSIF and SPEI tended to increase with longer timescales, except at the annual scale.

Interestingly, GOSIF for cultivated land exhibited a negative correlation with SPEI at the 1-, 3-, and 6-month scales. Conversely, the correlations between GOSIF and SPEI at the 6- and 9-month scales were more positive for shrubland compared to other land types. Additionally, the mean correlation coefficients between GOSIF and SPEI at different timescales were generally higher for grassland compared to the other three vegetation types.

### 3.4 Future projections of GOSIF and annual-scale SPEI in Xinjiang

**Figure 11:**
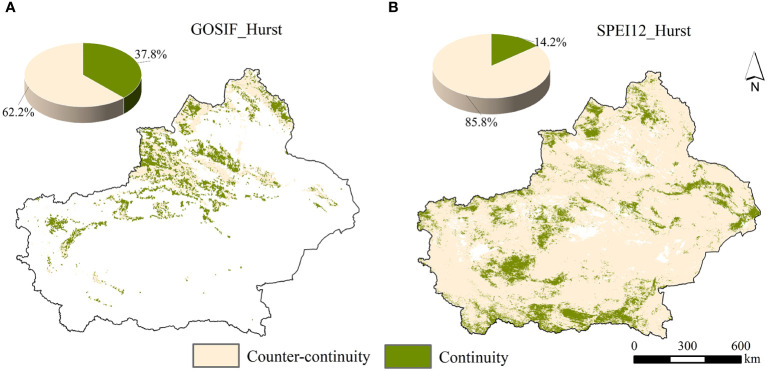
The spatial distribution of predicted sustained changes in GOSIF **(A)** and SPEI12 **(B)** in the future arid zone of Xinjiang.

The trends of GOSIF and SPEI in the study area were assessed using the Hurst index (**Figure 11**). It was found that the area of future Xinjiang vegetation GOSIF, with sustainability trends consistent with the past, accounted for 37.78% of the total area. This trend is projected to further increase in regions such as the Altai Mountains, near the Tian Shan Mountains, and in the oasis area of the northwestern Tarim Basin, while a decrease is anticipated near the Ili River Valley. Combining the Sen trend in GOSIF from 2001 to 2020 with the overall Hurst average suggests a potential decrease in GOSIF for vegetation in the future.

Furthermore, approximately 85.93% of Xinjiang’s area exhibited a future vegetation SPEI trend opposite to that of the past. Combining the 2001–2020 SPEI Sen and Hurst means (0.413) suggests a likelihood of increased aridity in Xinjiang in the future, with these areas primarily distributed in the Altai Mountains, near the Ili Valley, and in the central Tarim Basin.

## 4 Discussion

### 4.1 Comparison with relevant studies

Based on the analysis of GOSIF, vegetation index, and multiscale SPEI drought data from 2000 to 2020, this study extensively investigates the spatial and temporal variations of GOSIF and SPEI12, along with the sensitivity of GOSIF to multiscale SPEI. Additionally, it explores the response mechanism of GOSIF to drought in different vegetation types. Notably, the distribution of drought in Xinjiang exhibits significant regional differences attributed to its unique geographical location and climatic conditions. The study reveals that southern and eastern Xinjiang are predominantly affected by severe and moderate droughts, while mild droughts prevail in the west (**Figure 3A**). Cao et al. (2021) conducted an MK trend analysis of SPEI and found that the eastern part of Xinjiang was more arid than the western region. Based on the data such as measured precipitation and runoff, Wang (2023) constructed an aridity distribution map of Xinjiang, indicating that the southern border was more arid than the northern border and that the plains were more arid than the mountains. These conclusions are consistent with the results of this study. Such findings underscore the severity and complexity of the drought issue in Xinjiang. It is noteworthy that over the past 20 years, there has been a discernible downward trend in the degree of drought in the Xinjiang region, indicating an alleviation of the drought situation (Bai et al., 2019). This trend could be attributed to factors such as global climate change, water resource management strategies implemented in Xinjiang, and efforts toward ecological conservation measures.

The overall trend of SPEI spatial distribution is primarily characterized by a non-significant increase, particularly evident in the south and east (**Figure 5**). This observation further validates the trend of drought mitigation in Xinjiang and aligns with the perspectives of other scholars (Shi et al., 2002; Huang et al., 2020; Yu et al., 2023). Peng et al. (2018) highlighted an increasing trend in the frequency of droughts in the Northwest Arid Zone (including Xinjiang) from 1948 to 2012 based on the scPDSI index and copula method, which is slightly different from the results of this study. This is because the study period, selected drought indicators, and the methods of this study are different from ours. This discrepancy may also be attributed to the multitemporal scale nature of the SPEI drought index utilized in this study. The multiscalar characteristic of SPEI enables it to more accurately capture the complexity and dynamics of droughts (Cao et al., 2021).

The findings of this study demonstrate that GOSIF exhibits higher sensitivity in drought monitoring compared to traditional vegetation indices such as NDVI and EVI (**Figures 6**, **7**). GOSIF showed a more rapid and significant response to SPEI, both at the annual scale and during the growing season of a typical dry year. Similar observations were reported in northwest India, where Song et al. (2018) found that SIF responded to drought earlier than traditional vegetation indices like NDVI and EVI, facilitating the early monitoring of drought stress in wheat. Furthermore, Liu et al. (2018a) classified wheat plots into four different drought levels and observed that SIF remained sensitive and highly responsive to severe and extreme drought conditions, whereas NDVI exhibited sensitivity only to extreme drought.

While some studies have investigated the capability of GOSIF in drought monitoring and estimating total primary productivity (Jiao et al., 2019; Zhao and Wang, 2021), there remains a scarcity of research focusing on the response of GOSIF to drought across various vegetation types during typical dry years. In this study, we examined the characteristics of GOSIF response to drought in different vegetation types, including grassland, shrubland, and cultivated land, during the growing season of 2009 in the typically arid region of Xinjiang. Our findings revealed significant variations in the mean values of GOSIF–SPEI correlation among different vegetation types (**Figure 10**), consistent with previous research (Xu et al., 2018; Cao et al., 2022). These differences are primarily attributed to the adaptive capacity and physiological mechanisms of distinct vegetation types in response to drought stress.

### 4.2 Causes behind the changes in drought trends

**Figure 12:**
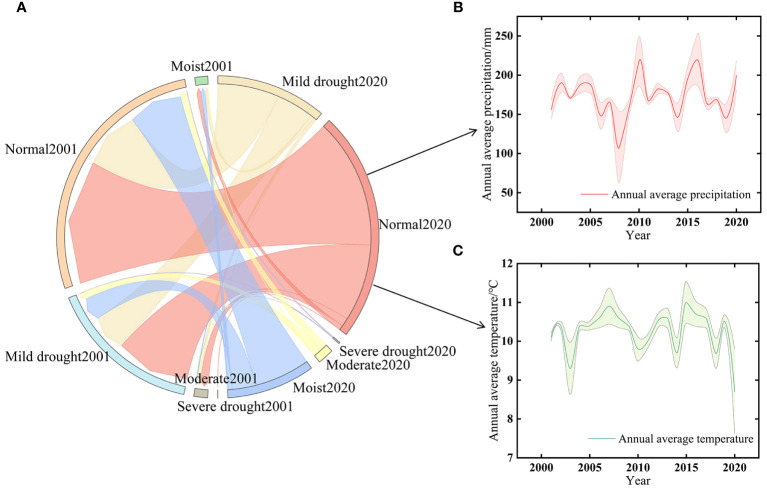
**(A)** The shift in aridity from 2001 to 2020. **(B)** The time series of mean annual precipitation from 2001 to 2020. **(C)** The time series of mean annual temperature from 2001 to 2020.

An in-depth study presented in this paper reveals that Xinjiang’s overall trend has slowly shifted toward increased moisture and reduced aridity over the past 20 years (**Figures 3C**, **12**). This change is influenced by a combination of factors. First, this may be related to the increase of water vapor content into Xinjiang due to global warming. Gao et al. (2023) systematically analyzed the spatial and temporal characteristics of water vapor content, precipitation, and other elements in the Central Asian arid zone from 1979 to 2018 based on reanalysis data and found that a tendency of an increase in water vapor content and precipitation exists in Xinjiang, which are 0.09 mm·(10a)^−1^ and 4.14 mm·(10a)^−1^, respectively. Additionally, this phenomenon has accelerated the rate of glacier ablation. Glaciers, serving as vital water sources in Xinjiang, play a crucial role in augmenting river water volume through the recharge of their meltwater (Lu et al., 2021).

As a consequence, Xinjiang has experienced a sustained period of relative water abundance in the last 30 years (Wang, 2023), creating favorable conditions for mitigating drought severity. Besides natural factors, anthropogenic activities driven by policy have also contributed positively to climate dynamics in Xinjiang. Since 1978, China has implemented many ecological restoration and protection measures. Among them, Xinjiang has completed a total of 15,171.54 km^2^ of artificial afforestation based on key projects such as the “Three Norths” protection forest, returning farmland to forests and grassland, and preventing and treating sand around the Tarim Basin, raising the forest coverage rate from 1.03% to 5.06% (https://t.m.china.com.cn/convert/c_vlEkj317.html). These efforts have led to increased vegetation cover, enhanced vegetation photosynthetic rates, and improved soil moisture conditions, consequently alleviating the extent of drought.

Nevertheless, despite evidence suggesting a trend toward warmer and wetter conditions in Xinjiang, this “warming and wetting” phenomenon remains quite restricted. The spatial distribution pattern of arid climates in Xinjiang has not undergone fundamental changes, and the arid zone’s natural environment continues to confront numerous challenges. Studies such as that by Cao et al. (2021) have indicated a potential drying trend in Northwest China in the future, while Li et al. (2021) and their team have projected more severe and prolonged droughts in Northwest China and even Central Asia. To gain a deeper understanding of the future trend of drought in Xinjiang, this paper employs the Hurst index for predictions. The results suggest that Xinjiang’s SPEI trend may encounter a more severe drought challenge ahead. This could be attributed to ongoing global warming, resulting in annual temperature rises in Xinjiang. Consequently, water dissipation from the ground and vegetation becomes easier over time. Additionally, indiscriminate afforestation in arid and semiarid areas may contribute to soil and water loss, exacerbating soil erosion and land degradation (Ma et al., 2023b). These effects not only impact agricultural production and the ecological environment but also could escalate the frequency of natural disasters such as sandstorms and dust storms (Li et al., 2021). As the core region of the “Belt and Road” initiative, studying drought characteristics in Xinjiang holds significant implications for ecological transformations, water resource management, and the sustainable development of human society in the region.

To summarize, a series of effective measures need to be implemented to address the ecological and environmental challenges faced by the arid areas of Xinjiang, such as vegetation degradation and soil erosion. For instance, tree planting activities can be carried out to enhance the water utilization efficiency of vegetation. Simultaneously, promoting water-saving irrigation techniques can significantly reduce water resource wastage. Additionally, strengthening research and application of soil water retention capacity is also crucial for addressing the drought problem. By implementing these measures, the ecological environment of Xinjiang can be gradually improved, and the sustainable development of the region can be promoted.

### 4.3 Evaluation of the potential of GOSIF in drought monitoring

GOSIF is a spectral signal emitted by chlorophyll molecules in chloroplasts when plants are exposed to light, directly reflecting the intensity and efficiency of photosynthesis. Contrastingly, the NDVI assesses vegetation growth by measuring the difference in the reflectance of red and near-infrared light, whereas the EVI considers atmospheric scattering and incorporates a blue band to improve the accuracy of monitoring vegetation growth. It has been shown that GOSIF in high vegetation cover areas of tropical rainforests more accurately reflects the real growth condition of vegetation than NDVI and EVI (Mohammed et al., 2019). Additionally, Liu et al. (2022a) found that GOSIF was more sensitive than NDVI and EVI to drought events for different vegetation types in China. Vegetation photosynthesis and key physiological processes are affected by drought (such as stomatal closure, reduced chlorophyll content, and slowed photosynthetic rate); however, these physiological changes are not immediately apparent in the spectral characteristics of the vegetation canopy, resulting in a delayed response to drought in the NDVI and EVI (Doughty et al., 2021). Therefore, the excellent performance of GOSIF makes it a powerful tool for drought monitoring and assessment.

The findings of this study highlight the significant role of GOSIF in drought monitoring, particularly in Xinjiang’s arid regions. The areas of high correlation between GOSIF and SPEI12 were mainly concentrated in the southern part of Tacheng, northern part of Ili, and, to a lesser extent, in the east-central part of the Tian Shan mountain range. This may be related to the higher dependence of vegetation growth status on water conditions. GOSIF and SPEI12 correlations were lowest at the edge of the Tarim Basin, mainly due to the wide distribution of arable land in the region, which supplies water to crops through artificial irrigation, resulting in vegetation that is insensitive to drought (Yuan et al., 2021). During a typical drought year, the mean correlation between GOSIF and the drought index of grassland was higher compared to forestland, shrubland, and cultivated land in Xinjiang. This prominence can be attributed to the characteristics of grassland, which typically possesses a shorter root system and lower water storage capacity, rendering it more susceptible to drought (Liu et al., 2022b). When drought occurs, the limited root system of grassland inhibits effective water absorption from the soil, leading to substantial impacts on vegetation’s physiological functions. Moreover, degraded grasslands often experience a significant loss of soil moisture, further diminishing their drought tolerance and weakening their photosynthetic capacity. Consequently, the drought’s impact on grassland ecosystems is exacerbated (Liang et al., 2021). Cultivated land exhibits a less pronounced response to drought sensitivity, primarily due to human activities significantly influencing its water use efficiency. Zhu et al. (2021) showed that the effect of drought on the growing season EVI of irrigated farmland was 19.98% lower than that of natural vegetation EVI. This is mainly because irrigation, fertilizer application, and advanced planting techniques effectively improve the water absorption capacity of the crop root system, thus mitigating the effects of drought on crops (Liu et al., 2023).

In addition, the study showed that the GOSIF of different vegetation types in the arid zone of Xinjiang showed a certain pattern of response to SPEI, and the results of the study showed that, among them, the GOSIF of different vegetation types in the growing season had the most significant response to the SPEI at the 9-month scale (**Figure 9**), followed by the SPEI at the 6-month scale, which suggests that photosynthesis in Xinjiang’s arid regions is more influenced by medium- and long-term drought stresses (Wang, 2023). It is noteworthy that glacial snowmelt in Xinjiang’s arid regions, particularly in the Tianshan Mountains and the Kunlun Mountains, supplies ample water resources for the local vegetation, thereby mitigating the impacts of drought to some degree. This observation aligns with the conclusions drawn by Liu et al. (2022a) and Sun et al. (2021). Moreover, several studies have indicated that various vegetation types in Xinjiang exhibit a degree of adaptability and resilience to drought stress. These plants adjust their growth strategies and enhance the water absorption capacity of their root systems to adapt to drought conditions.

### 4.4 Limitation analysis

The GOSIF data used in this study were obtained via a data-driven approach using OCO-2, MODIS, and meteorological reanalysis data. Although the data are characterized by high spatial and temporal resolutions, certain errors and uncertainties are introduced during data acquisition and processing due to external factors (such as light conditions, precipitation, and soil moisture) and different selection methods. Moreover, it is important to recognize the complexity and variability of drought issues, often challenging for a single monitoring indicator and methodology to fully capture. Therefore, future research should combine multiple data sources and research tools, such as multisource remote sensing data, ground observation data, radar data, and model simulation methods, to construct a more accurate drought monitoring and simulation system. Simultaneously, we need to strengthen our research on the mechanisms of drought occurrence to provide a theoretical basis for the formulation of effective drought strategies.

## 5 Conclusions

From 2001 to 2020, we investigated the correlation between GOSIF, NDVI, and EVI with SPEI12 of vegetation and the variation in drought class in Xinjiang. This analysis utilized the Sen+Mann–Kendall trend analysis, SAI, Pearson correlation, and Hurst index analysis methods applied to time-series data. Furthermore, we examined the response relationship between vegetation GOSIF and SPEI at different scales during the growing season of 2009, a representative drought year in the study area. Finally, we forecasted the future trends of vegetation GOSIF and SPEI in Xinjiang. The main conclusions are as follows:

1. During the period from 2001 to 2020, the study area experienced predominantly moderate drought conditions, primarily observed in the Tarim Basin, Junggar Basin, and near the Altay Mountains in Xinjiang. Regions classified as wet or normal showed an increasing trend in drought occurrence.
2. GOSIF exhibited a higher correlation with SPEI12 compared to NDVI or EVI, with a maximum correlation coefficient of 0.906, indicating its superior efficiency in drought monitoring. Among different vegetation types, grassland GOSIF showed the highest sensitivity to drought.
3. Vegetation GOSIF exhibited the highest correlation with SPEI09, indicating greater sensitivity, followed by SPEI06, and the lowest correlation with SPEI01. Among various vegetation types, grassland GOSIF showed the highest correlation coefficient with SPEI09.
4. Combined with the trend of SPEI during 2001–2020 and the average Hurst mean value of SPEI at 0.413, our findings suggest that drought in Xinjiang will likely increase in the future, primarily concentrated in the Altai Mountains, near the Ili Valley, and in the central part of the Tarim Basin.

## Data Availability

The original contributions presented in the study are included in the article/supplementary material. Further inquiries can be directed to the corresponding author.

## References

[B1] Adila A. Zan M. (2022). Analysis of drought change in Xinjiang based on SPEI. J. Anhui Agric. Sci. 50, 178–183. doi: 10.3969/j.Issn.0517-6611.2022.11.046

[B2] AghaKouchak A. Farahmand A. Melton F. S. Teixeira J. Anderson M. C. Wardlow B. D.. (2015). Remote sensing of drought: progress, challenges and opportunities. Rev. Geophys. 53, 452–480. doi: 10.1002/2014RG000456

[B3] Anderson M. C. Hain C. Wardlow B. Pimstein A. Mecikalski J. R. Kustas W. P. (2011). Evaluation of drought indices based on thermal remote sensing of evapotranspiration over the Continental United States. J. Clim. 24, 2025–2044. doi: 10.1175/2010JCLI3812.1

[B4] Aulia M. R. Liyantono Setiawan Y. Fatikhunnada A. (2016). Drought detection of west java’s paddy field using MODIS EVI satellite images (case study: rancaekek and rancaekek wetan). Proc. Environ. Sci. 33, 646iron. doi: 10.1016/j.proenv.2016.03.119

[B5] Bai Q. Yan P. Cai D. Jin H. Feng G. Zhang T. (2019). Inter-decadal change characteristics of different grades drought in northwest China in recent 56 years. J. Arid Meteorol. 37, 722–728. doi: 10.11755/j.issn.1006-7639(2019)-05-0722

[B6] Camps-Valls G. Campos-Taberner M. Moreno-Martínez Á. Walther S. Duveiller G. Cescatti A.. (2021). A unified vegetation index for quantifying the terrestrial biosphere. Sci. Adv. 7, eabc7447. doi: 10.1126/sciadv.abc7447 33637524 PMC7909876

[B7] Cao S. He Y. Zhang L. Chen Y. Yang W. Yao S.. (2021). Spatiotemporal characteristics of drought and its impact on vegetation in the vegetation region of Northwest China. Ecol. Indic. 133, 108420. doi: 10.1016/j.ecolind.2021.108420

[B8] Cao Y. Huang Z. Xu X. Chen S. Wang Z. Feng H.. (2022). Responses of solar-induced chlorophyll fluorescence to meteorological drought across the Loess Plateau, China. Chin. J. Appl. Ecol. 33, 457–467. doi: 10.13287/j.1001-9332.202202.011 35229520

[B9] Cao Y. Si W. Du Z. Liang H. Lei T. Sun B.. (2023). Changes in GPP of China during the typical drought years from 1982 to 2017. Arid zone Geogr. 46, 1577–1590. doi: 10.12118/j.issn.1000-6060.2023.078

[B10] Chen F. Yuan Y. Zhang T. Shang M. Yu S. Fan Z. (2015). Long – term drought severity variations in the northern Altay Mountains and its linkages to the Irtysh River streamflow variability. J. Arid Land Resources Environ. 29, 93–98. doi: 10.13448/j.cnki.jalre.2015.263

[B11] Didan K. (2015). Data from: MODIS/Terra Vegetation Indices Monthly L3 Global 1km SIN Grid V006. NASA EOSDIS 660 Land Processes DAAC. doi: 10.5067/MODIS/MOD13A3.006

[B12] Doughty R. Köhler P. Frankenberg C. Magney T. S. Xiao X. Qin Y.. (2019). TROPOMI reveals dry-season increase of solar-induced chlorophyll fluorescence in the Amazon Forest. Proc. Natl. Acad. Sci. U. S. A. 116, 22393–22398. doi: 10.1073/pnas.1908157116 31611384 PMC6825294

[B13] Doughty R. Xiao X. Köhler P. Frankenberg C. Qin Y. Wu X.. (2021). Global-scale consistency of spaceborne vegetation indices, chlorophyll fluorescence, and photosynthesis. J. Geophys. Res. Biogeosci. 126, e2020. doi: 10.1029/2020JG006136

[B14] Fang W. Huang S. Huang Q. Huang G. Wang H. Leng G.. (2019). Bivariate probabilistic quantification of drought impacts on terrestrial vegetation dynamics in mainland China. J. Hydrol. 577, 123980. doi: 10.1016/j.jhydrol.2019.123980

[B15] Gao J. Zhao Y. Yao J. Dilinuer T. Wang M. (2023). Spatial and temporal evolution of atmospheric moisture cycle elements in the arid zone of Central Asia in the context of climate change. Arid Zone Res. 39, 1371–1384. doi: 10.13866/j.azr.2022.05.04

[B16] Guanter L. Alonso L. Gómez-Chova L. Amorós-López J. Vila J. Moreno J. (2007). Estimation of solar-induced vegetation fluorescence from space measurements. Geophys. Res. Lett. 34, L08401. doi: 10.1029/2007GL029289

[B17] Han W. Guan J. Zheng J. Liu Y. Ju X. Liu L.. (2023). Probabilistic assessment of drought stress vulnerability in grasslands of Xinjiang, China. Front. Plant Sci. 14. doi: 10.3389/fpls.2023.1143863 PMC10062607 37008478

[B18] Huang J. Zhang Y. Wang M. Wang F. Tang Z. He H. (2020). Spatial and temporal distribution characteristics of drought and its relationship with meteorological factors in Xinjiang in last 17 years. Acta Ecologica Sinica. 40, 1077–1088. doi: 10.5846/stxb201810302341

[B19] Janni M. Maestri E. Gullì M. Marmiroli M. Marmiroli N. (2024). Plant responses to climate change, how global warming may impact on food security: a critical review. Front. Plant Sci. 14. doi: 10.3389/fpls.2023.1297569 PMC10796516 38250438

[B20] Jiang W. Wang L. Zhang M. Yao R. Chen X. Gui X.. (2021). Analysis of drought events and their impacts on vegetation productivity based on the integrated surface drought index in the Hanjiang River Basin, China. Atmos. Res. 254, 105536. doi: 10.1016/j.atmosres.2021.105536

[B21] Jiao W. Chang Q. Wang L. (2019). The sensitivity of satellite solar-induced chlorophyll fluorescence to meteorological drought. Earths Future 7, 558–573. doi: 10.1029/2018EF001087

[B22] Jung-Ching K. Carla S. S. F. Georgia D. Pan H. Marlon P. V. Zahra B. K. K. (2023). Predicting agricultural drought indicators: ML approaches across wide-ranging climate and land use conditions. Ecol. Indic. 154, 110524. doi: 10.1016/j.ecolind.2023.110524

[B23] Kaushik P. R. Ndehedehe C. E. Kalu I. Burrows R. M. Noll M. R. Kennard M. J. (2023). Identifying potential hotspots of groundwater-climate interaction in the Great Artesian Basin, Australia. Ecol. Infor. 78, 102354. doi: 10.1016/j.ecoinf.2023.102354

[B24] Khalil G. Jahangir M. Laleh G. R. (2024). Annual growth of Fagus orientalis is limited by spring drought conditions in Iran’s Golestan Province. J. For. Res. 35, 132–146. doi: 10.1007/s11676-023-01674-7

[B25] Kong J. Zan M. Wang X. Yang X. (2022). Spatiotemporal patterns of vegetation water use efficiency and its influencing factors in Manas River Basin, Xinjiang. J. Water Resour. Water Eng. 33, 196–203. doi: 10.11705/j.issn.1672-643X.2022.06.25

[B26] Li H. Li Z. Chen Y. Liu Y. Hu Y. Sun F.. (2021). Projected meteorological drought over Asian drylands under different CMIP6 scenarios. Remote Sens. 13, 4409. doi: 10.3390/rs13214409

[B27] Li Y. Ding J. Zhang J. Wu P. (2019). Response of vegetation cover to drought in the northern slopes of the Tianshan Mountain during 2001 – 2015 based on land-use and land-cover change. Acta Ecologica Sin. 39, 6206–6217. doi: 10.5846/stxb201811112442

[B28] Li X. Xiao J. (2019). A Global, 0.05-degree product of solar-induced chlorophyll fluorescence derived from OCO-2, MODIS, and reanalysis data. Remote Sens. 11, 517–541. doi: 10.3390/rs11050517

[B29] Liang M. Cao R. Di K. Han D. Hu Z. (2021). Vegetation resistance and resilience to a decade-long dry period in the temperate grasslands in China. Ecol. Evol. 11, 10582–10589. doi: 10.1002/ece3.7866 34367598 PMC8328410

[B30] Liu Y. Li C. Liu Z. Deng X. (2016a). Assessment of spatio-temporal variations in vegetation cover in Xinjiang from 1982 to 2013 based on GIMMS-NDVI. Acta Ecologica Sin. 36, 6198–6208. doi: 10.11707/j.1001-7488.20151005

[B31] Liu X. Pan Y. Zhu X. Yang T. Bai J. Sun Z. (2018b). Drought evolution and its impact on the crop yield in the North China Plain. J. Hydrol. 564, 984–996. doi: 10.1016/j.jhydrol.2018.07.077

[B32] Liu Y. Ren H. Hu T. Yang P. Basan S. Zhang W.. (2022b). Spatiaotemporal dynamics of NDVI of grassland and its response to multi–scale drought in China. Res. Soil Water Conserv. 29, 153–161. doi: 10.13869/j.cnki.rswc.2022.01.017

[B33] Liu Y. Wang H. Ju W. (2016b). Characteristics of drought impact on forest productivity in Jiangxi Province. Nat. Hazards. 25, 67–77. doi: 10.13577/j.jnd.2016.0308

[B34] Liu L. Yang X. Zhou H. Liu S. Zhou L. Li X.. (2018a). Relationship of root zone soil moisture with solar-induced chlorophyll fluorescence and vegetation indices in winter wheat: A comparative study based on continuous ground-measurements. Ecol. Indic. 90, 9–17. doi: 10.1016/j.ecolind.2018.02.048

[B35] Liu Z. Yao M. Yang Z. Zhang Q. Xu X. (2023). Alleviation effect of fertilization and irrigation on summer maize drought and its influencing factors based on meta-analysis. Chin. J. Agrometeorology 44, 361–371. doi: 10.3969/j.issn.1000-6362.2023.05.002

[B36] Liu Q. Zhang F. Zhao X. (2022a). The superiority of solar-induced chlorophyll fluorescence sensitivity over other vegetation indices to drought. J. Arid Environ. 204, 104787. doi: 10.1016/j.jaridenv.2022.104787

[B37] Lu B. Sun H. Jiang Q. Cao J. Lan X. Zhang L.. (2021). Spatiotemporal variation Characteristics of the water budget and in Xinjiang during the latest 53 years. Arid Zone Res. 38, 1579–1589. doi: 10.13866/j.azr.2021.06.10

[B38] Ma Q. Su Y. Niu C. Hu T. Luo X. Tai X.. (2023b). Tree mortality during long-term droughts is lower in structurally complex forest stands. Nat. Commun. 14, 7467–7467. doi: 10.1038/s41467-023-43083-8 37978191 PMC10656564

[B39] Ma J. Zhang C. Li S. Yang C. Chen C. Yun W. (2023a). Changes in vegetation resistance and resilience under different drought disturbances based on NDVI and SPEI time series data in Jilin Province, China. Remote Sens. 15, 3280. doi: 10.3390/rs15133280

[B40] Marzieh M. Rassoul A. Z. (2023). Soil erosion prediction using Markov and CA-Markov chains methods and remote sensing drought indicators. Ecol. Inf. 78, 102386. doi: 10.1016/j.ecoinf.2023.102386

[B41] Mohammed G. H. Colombo R. Middleton E. M. Rascher U. van der Tol C. Nedbal L.. (2019). Remote sensing of solar-induced chlorophyll fluorescence (SIF) in vegetation: 50 years of progress. Remote Sens. Environ. 231, 111177. doi: 10.1016/j.rse.2019.04.030 33414568 PMC7787158

[B42] Peng Y. Xia J. Zhang Y. Zhan C. Qiao Y. (2018). Comprehensive assessment of drought risk in the arid region of Northwest China based on the global Palmer drought severity index gridded data. Sci. Total Environ. 627, 951–962. doi: 10.1016/j.scitotenv.2018.01.234 29426220

[B43] Piao S. Zhang X. Chen A. Liu Q. Lian X. Wang X.. (2019). The impacts of climate extremes on the terrestrial carbon cycle: A review. Sci. China Earth Sci. 62, 1551–1563. doi: 10.1007/s11430-018-9363-5

[B44] Qi X. Miao C. Wang H. (2023). Detecting Response of vegetation photosynthetic to meteorological drought based on solar-induced chlorophyll fluorescence. China Agric. Meteorol. 44, 133–143. doi: 10.3969/j.issn.1000-6362.2023.02.005

[B45] Ruzi H. Liu H. Hoyhazi E. (2023). Trend analysis of meteorological drought in Changji region from 1961 to 2020 based on standardised precipitation index. Water Sav. Irrig. 5), 84–95. doi: 10.12396/jsgg.2022370

[B46] Shi Y. Shen Y. Hu R. (2002). Preliminary study on signal, Impact and foreground of climatic shift from warm-dry to warm-humid in northwest China. J. glaciology geocryology 24, 219–226. doi: 10.7522/j.issn.1000-0240.2002.0044

[B47] Song L. Guanter L. Guan K. You L. Huete A. Ju W.. (2018). Satellite sun-induced chlorophyll fluorescence detects early response of winter wheat to heat stress in the Indian Indo-Gangetic Plains. Glob. Change Biol. 24, 4023–4037. doi: 10.1111/gcb.14302 29749021

[B48] Song J. Xu C. Yang Y. Zhang X. Li X. (2019). Temporal and spatial variation Characteristics of evapotranspiration and dry–wet climate in Xinjiang based on MODIS16. Res. Soil Water Conserv. 26, 210–214+221+2. doi: 10.13869/j.cnki.rswc.2019.05.031

[B49] Sun S. Du W. Song Z. Zhang D. Wu X. Chen B.. (2021). Response of gross primary productivity to drought timescales across China. J. Geophys. Res. Biogeosci. 126, e2020. doi: 10.1029/2020JG005953

[B50] Tong S. Lai Q. Zhang J. Bao Y. Lusi A. Ma Q.. (2018). Spatiotemporal drought variability on the Mongolian Plateau from 1980-2014 based on the SPEI-PM, intensity analysis and Hurst exponent. Sci. Total Environ. 615, 1557–1565. doi: 10.1016/j.scitotenv.2017.09.121 28923710

[B51] Tu Y. Jiang L. Liu R. Xiao Z. Min J. (2021). Spatiotemporal changes of vegetation NDVI and its driving forces in China during 1982–2015. Trans. Chin. Soc. Agric. Eng. 37, 75–84. doi: 10.11975/j.issn.1002-6819.2021.22.009

[B52] Varela V. Vlachogiannis D. Sfetsos A. Karozis S. Politi N. P. F. Giroud F. (2019). Projection of forest fire danger due to climate change in the French Mediterranean region. Sustainability 11, 4284. doi: 10.3390/su11164284

[B53] Wang J. (2023). The distribution and evolution of Xinjiang Arid Zone under climate warming and humidification. Arid Environ. Monit. 37, 15–21.

[B54] Wang S. Wang J. Zhang Q. Li Y. (2020). Applicability evaluation of drought indices in Northern China and the reasons for their differences. Plateau Meteorol. 39, 628–640. doi: 10.7522/j.issn.1000-0534.2019.00049

[B55] Wu X. Duan C. Mayila B. Zhang J. Zhang T. (2022). Analysis of the temporal-spatial variation characteristics of drought in the Xinjiang based on the meteorological drought comprehensive index. Arid Zone Res. 39, 75–83. doi: 10.13866/j.azr.2022.01.08

[B56] Xia H. Zhao X. Zhao W. Jiao W. (2023). Data from: High-Resolution SPEI Dataset for Drought Monitoring and Impact Analysis in Mainland China from 2001 to 2020. V2. National Ecosystem Data Bank. doi: 10.57760/sciencedb.ecodb.00090

[B57] Xu H. Wang X. Zhao C. Shan S. Guo J. (2021a). Seasonal and aridity influences on the relationships between drought indices and hydrological variables over China. Weather Clim. Extremes 34, 100393. doi: 10.1016/j.wace.2021.100393

[B58] Xu H. Wang X. Zhao C. Yang X. (2018). Diverse responses of vegetation growth to meteorological drought across climate zones and land biomes in northern China from 1981 to 2014. Agric. For. Meteorol. 262, 1–13. doi: 10.1016/j.agrformet.2018.06.027

[B59] Xu H. Wang X. Zhao C. Yang X. (2021b). Assessing the response of vegetation photosynthesis to meteorological drought across Northern China. Land Degrad. Dev. 32, 20–34. doi: 10.1002/ldr.3701

[B60] Yang S. Meng D. Li X. Wu X. (2018). Multi-scale responses of vegetation changes relative to the SPEI meteorological drought index in North China in 2001–2014. Acta Ecologica Sin. 38, 1028–1039. doi: 10.5846/stxb201611242398

[B61] You M. He Z. Zhang L. Yang M. Pi G. (2022). Characteristics of agricultural and meteorological drought in Guizhou Province and their response relationship. J. Soils Water Conserv. 36, 255–264. doi: 10.13870/j.cnki.stbcxb.2022.05.032

[B62] Yu J. Zhang J. Zhang M. Yu R. (2023). Spatial and temporal evolution of drought in Xinjiang based on the standardised precipitation evapotranspiration index. Agric. Res. Arid Areas 41, 275–288. doi: 10.7606/J.ISSN.1000-7601.2023.04.29

[B63] Yuan X. Peng Z. Liu X. (2021). Different time-scale responses of vegetation to the SPEI drought index in Xinjiang. Desert Oasis Meteorology 15, 129–136. doi: 10.12057/j.issn.1002-0799.2021.03.017

[B64] Zhang G. Su X. Hao L. Wu H. (2019). Response of vegetation to drought based on NDVI and scPDSI data sets from 1982 to 2015 across China. Trans. Chin. Soc Agric. Eng. 35, 145–151. doi: 10.11975/j.issn.1002-6819.2019.20.018

[B65] Zhao Z. Wang K. (2021). Capability of existing drought indices in reflecting agricultural drought in China. J. Geophysical Research: Biogeosciences 126, e2020JG006064. doi: 10.1029/2020JG006064

[B66] Zhao F. Wang L. Ma Y. Jiang R. (2024). Response of vegetation growth condition to meteorological drought in Shanxi Province from 2000 to 2020. Remote Sens. Nat. Resour., 1–11. doi: 10.6046/zrzyyg.2023226

[B67] Zhong R. Yan K. Gao S. Yang K. Zhao S. Ma X.. (2023). Response of grassland growing season length to extreme climatic events on the Qinghai-Tibetan Plateau. Sci. Total Environ. 909, 168488–168488. doi: 10.2139/ssrn.4542633 37972770

[B68] Zhou Y. Gui Y. Zhang Q. Chen M. Liu Y. (2024). The study on spatial distribution of water ecological environment carrying capacity during extreme drought conditions. Sci. Rep. 14, 11986–11986. doi: 10.1038/s41598-024-62856-9 38796635 PMC11128009

[B69] Zhou Z. Shi H. Fu Q. Li T. Gan T. Liu S. (2020). Assessing spatiotemporal characteristics of drought and its effects on climate-induced yield of maize in Northeast China. J. Hydrol. 588, 12509. doi: 10.1016/j.jhydrol.2020.125097

[B70] Zhu X. Liu Y. Xu K. Pan Y. (2021). Effects of drought on vegetation productivity of farmland ecosystems in the drylands of Northern China. Remote Sens. 13, e1179. doi: 10.3390/rs13061179

[B71] Zuo D. Cai S. Xu Z. Peng D. Kan G. Sun W.. (2019). Assessment of meteorological and agricultural droughts using *in-situ* observations and remote sensing data. Agric. Water Manage. 222, 125–138. doi: 10.1016/j.agwat.2019.05.046

